# Developing Balanced Quality Indicators for Monitoring Virtual Care in Ambulatory Care Environments: Modified Delphi Panel Process

**DOI:** 10.2196/38657

**Published:** 2025-06-16

**Authors:** Samuel Petrie, Oluwatoni Makanjuola, Celia Laur, Emeralda Burke, Patricia Rios, Onil Bhattacharyya, Geetha Mukerji

**Affiliations:** 1 Implementation Science Team Research, Innovation, and Discovery Nova Scotia Health Halifax, NS Canada; 2 Department of Community Health and Epidemiology Faculty of Medicine Dalhousie University Halifax, NS Canada; 3 Women's College Hospital Institute for Health System Solutions and Virtual Care Women's College Hospital Toronto, ON Canada; 4 Institute of Health Policy, Management and Evaluation Dala Lana School of Public Health University of Toronto Toronto, ON Canada; 5 Ontario Breast Screening Program Ontario Health Toronto, ON Canada; 6 Department of Family and Community Medicine Temerty Faculty of Medicine University of Toronto Toronto, ON Canada; 7 Department of Medicine Temerty of Faculty of Medicine University of Toronto Toronto, ON Canada

**Keywords:** virtual care, quality, quintuple aim, Delphi panel, quality indicators, performance assessment

## Abstract

**Background:**

While the volume of virtual visits increased with the COVID-19 pandemic, little is still known regarding the quality of virtual visits. Furthermore, there is limited guidance on how best to evaluate the quality of virtual care initiatives.

**Objective:**

The objective of this study was to curate a balanced set of quality indicators grounded in the Quintuple Aim quality framework and the National Academy of Medicine domains of quality, including sustainability, to monitor the quality of virtual care in ambulatory environments.

**Methods:**

Phase 1 involved curating a list of ambulatory virtual care quality indicators from published and grey literature, along with knowledge user feedback generated through a pre-Delphi survey; these indicators were mapped and categorized to the Quintuple Aim and National Academy of Medicine (NAM) Quality Domains. In phase 2, a 19-member pan-Canadian panel was convened as part of a 3-round modified Delphi panel process to rate and rank the quality indicators. The panel included clinicians, individuals with lived experience with the health care system, policy makers, academics, and administrators. Panelists rated indicators using the Agency for Healthcare Research and Quality measure attributes on a 9-point Likert scale in round 1, followed by a virtual Delphi panel meeting to discuss indicators before round 2 of re-rating, then a final round 3 of ranking which assessed the importance of indicators within each quality domain and subdomain. To address gaps in the literature, novel quality indicators of virtual care were identified and discussed in panel discussions, patient group consultations, and a pre-Delphi survey. For indicator advancement during the rating exercises, 75% or more of the panelists’ responses in the top tertile (scores of 7-9) with a median composite score of 7 or greater was required.

**Results:**

There were 140 quality indicators identified in the literature which progressed through the 3 Delphi panel rounds. There was minimal attrition among Delphi panel members (17/19, 89% participated in all 3 rounds). After round 3, 25 indicators were included in the final scorecard. Aligned with the Quintuple Aim, there are 13 quality indicators on patient experience, 6 on provider experience, 2 on population health, 2 on health equity, and 2 on health system costs.

**Conclusions:**

A balanced set of 25 quality indicators of ambulatory virtual care was developed based on literature and consensus building from knowledge users across the health system. This curated set of indicators will support more comprehensive evaluations of virtual care. Organizations can use the set of indicators as part of a scorecard to compare across jurisdictions, identify priority areas, and ensure their virtual care initiatives are delivering high-quality care based on multiple domains of quality.

## Introduction

The rapid adoption of virtual care across all levels of the health system was driven by the COVID-19 pandemic [1]. Virtual care is defined as any interaction between patients and or caregivers and their health care providers (or “circle of care”) that occurs remotely and is facilitated through digital communication or other information technologies [2]. In Ontario, Canada, virtual visits rose from 146,014 in 2016 to 4,927,830 in 2020. Furthermore, virtual visits in rural regions increased 969% from 27,145 in 2016 to 290,401 in 2020 [3]. As COVID-19 pandemic measures have subsided, many temporary virtual service programs have become integrated into the standard of care as part of hybrid models of care delivery [4]. These hybrid care models combine in-person care with virtual care, such as remote monitoring or video visits in between for symptom monitoring or assessment [5]. In part, the adoption of this hybrid model integrating both in-person and virtual care has been supported by the ease of access, remuneration models, and preferences of many patients and health care professionals [5-7].

Despite the extensive uptake of virtual care, there is limited guidance on how to best evaluate the quality of virtual care visits. Virtual care evaluations conducted during the pandemic largely focused on narrow domains of quality, such as patient satisfaction, with limited assessment on other relevant quality domains, including the impact on equity and or population health [8-10]. Further, existing evaluation frameworks do not address all elements across the continuum of the implementation and evaluation of a prospective virtual care program [11]. Available quality frameworks are also siloed in their considerations of quality and lack an integrated approach to assessing virtual care.

While virtual care can be beneficial to improve access and respect the preferences of patients and or providers, it might not always be appropriate [11]. There is still some hesitation around virtual care, as this modality is seen to limit the comprehensiveness of care, such as the inability to perform a physical examination [12]. Patients have also reported negative experiences, such as receiving “bad news” through virtual meetings, or accessing test results before they have been reviewed or interpreted by their provider [13]. Assessing the quality of virtual care is integral to ascertain, as it can influence the effectiveness of treatment and adherence to provider-recommended care plans [14].

To address this gap, a balanced scorecard for virtual care could provide a set of quality indicators to guide performance assessments across broad quality domains [15,16]. Quality indicators are standardized, evidence-based measures that can be used to track and compare health outcomes and performance over time and across different organizations [17]. A balanced scorecard (BSC) is a tool used by organizations to strategize about meeting their mandates and goals and has been used in health care [18,19].

Our objective was to develop a set of broad quality indicators that could be used as part of a balanced scorecard across the Quintuple Aim to evaluate the quality of virtual care. The Quintuple Aim is a framework that aims to improve health system performance by simultaneously addressing the patient and provider experience, and health of populations while reducing per capita costs and addressing equity [20]. To develop the scorecard, a modified Delphi consensus process was used due to the heterogeneity of opinions regarding quality indicators of virtual care.

By grounding the set of quality indicators on the Quintuple Aim framework [20], a balanced scorecard assessing the quality of virtual care could allow for a nuanced understanding of how these intersecting aspects impact the quality of care [21].

## Methods

### Overview

This study was conducted in 2 phases. Phase 1 was used to generate a candidate list of virtual care indicators, followed by phase 2, which involved a modified Delphi panel process to refine and curate a balanced set of quality indicators for virtual care. The ACrutate COnsensus Reporting Document (ACCORD) checklist [22] (Table S2 in Multimedia Appendix 2) was used to report the findings of the modified Delphi process [23] (Table S2 in Multimedia Appendix 2). The study was not registered. A steering committee of researchers, clinicians, a project manager, and a person with lived experience oversaw the administration and execution of the scoping review and Delphi process.

### Phase 1: Generating a Candidate List of Virtual Care Indicators

#### Overview

A rigorous scoping review that included published and gray literature guided by the Joanna Briggs Institute (JBI) Manual for Evidence Synthesis [24] was used to generate the candidate list of quality indicators of virtual care. Scoping review results are described elsewhere [21]. In brief, 5 databases (MEDLINE, Embase, PsycINFO, Cochrane Library, and JBI) and grey literature sources (11 websites, 3 search engines) were searched that analyzed virtual care in ambulatory settings. There were 13,504 citations that were double-screened resulting in 631 full-text articles, 66 of which were included. Papers were included if they explicitly discussed quality assessments of virtual care in ambulatory care environments. Extracted indicators (N=140 quality indicators) from published and grey literature were then aligned to 2 quality frameworks: the Quintuple Aim and the National Academies of Medicine (NAM) domains of quality of care. Extracted indicators within the provider and patient experience of the Quintuple Aim were further subdivided into the NAM quality domains, which include safety, effectiveness, efficiency, patient-centeredness, timeliness, and equity [25]. Composite indicators (indicators which addressed 1 or more of the NAM subdomains) were also included in the patient and provider experience metrics. The steering committee reviewed the frameworks and added the domain of sustainability to the structured extraction to ensure consideration of long-term quality was included. In this context, sustainability refers to the degree to which virtual care is integrated into ongoing organizational systems, ensuring gains are maintained. The codebook used to map the extracted indicators is described elsewhere [21]. All modalities of virtual care were included, such as videoconferencing, remote monitoring, and patient portals.

Following the extraction and mapping exercises, the steering committee reviewed indicators for completeness and redundancy. Any indicator that did not stand alone as a quality measure, was irrelevant, difficult to interpret, or duplicative in nature, was removed.

#### Pre-Delphi Survey

A pre-Delphi survey was conducted via email using the REDCap (Research Electronic Data Capture; Vanderbilt University) survey platform to assess the completeness of extracted indicators and the appropriateness of coding the indicators into the Quintuple Aim and NAM quality frameworks. Feedback on the candidate list of indicators generated from the scoping review was collected, perceived gaps were identified, new indicators could be suggested, and potential panel members could be nominated. This survey was sent to all Canadian Network for Digital Health Evaluation (CNDHE) members. Survey responses were reviewed by the steering committee and used to inform the inclusion of novel indicators before the round 1 rating. The remaining indicators were categorized to each quality domain for rating by the Delphi panel.

### Phase 2: Modified Delphi Panel Process

#### Overview

In this modified Delphi process, panelists rated each indicator in round 1, attended a virtual panel meeting in round 2, followed by a rerating of indicators that did not meet consensus, and a final round of ranking in round 3. The modified Delphi process is a well-established, consensus-building practice wherein content experts rate and rank specific indicators across 2 or more rounds according to their relevance to the issue in question [26]. It is an iterative, multistage process designed to combine individual opinion into group consensus and is most appropriate where there is little agreement regarding the method of appropriate action [27], such as with virtual care, which may have different meanings to different individuals, (eg, patients or providers, and rural or urban populations [28]). In addition, anonymity in voting allows for equal weighting of all perspectives (decreasing power differentials, such as between policy decision makers and partners with lived experiences), and the feedback sessions allow for discussion, clarification, and justification for decisions [29,30]. The Delphi panel was selected through a nomination process from the pre-Delphi survey, an invitation, and a knowledge user mapping exercise. This pan-Canadian Delphi process was conducted between June 2023 and February 2024.

#### Co-Design With Persons With Lived Experience

The person with lived experience perspective was critical in creating a scorecard that reflects the priorities and needs of patients when accessing care virtually. One of our co-investigators and steering committee members is a person with lived experience, who informed the study design, including the scoping review and Delphi process. The project was also presented to a patient partner group at Women’s College Hospital (the Community Liaison Advisory Committee [CLAC]) to solicit feedback on our overall approach and our plans for seeking person with lived experience input on the Delphi panel. The list of indicators was shared with the CLAC to gather feedback on potential gaps in the list and new indicators.

#### Training With Panelists

Before the first round of the Delphi, panelists were invited to an online training workshop where steering committee members explained the methodology and rationale for the project. The Agency for Healthcare Research and Quality (AHRQ) quality measures were described, as well as their functional meaning when rating and ranking. This level-setting exercise ensured all panel members were comfortable with the rating and ranking process, and any questions about the project or process could be addressed. This workshop also encouraged respectful collaboration given the power differentials within the panel. All participants, particularly persons with lived experiences, were provided the opportunity for further support if requested.

#### Delphi Round 1

For round 1, the list of candidate indicators from phase 1 was sent to panel members through REDCap [31], a secure survey software used in all rounds. To standardize the presentation of indicators, minor revisions of the wording of the indicators were conducted to be measurable as an indicator when necessary, including transformation into the numerator and or denominator when not available. To ensure consistency across indicators, standardization of nomenclature from virtual care synonyms (ie, telemedicine) to “virtual care” and “health care providers” to “clinician” was transformed, when appropriate. Panelists were asked to rate each indicator against the 4 AHRQ quality measures attributes on a 9-point Likert scale, where 1 indicates strong disagreement and 9 indicates strong agreement. Panelists’ ratings were equally weighted across the AHRQ’s measures:

- Scientific soundness: clinical logic (the evidence supporting the indicator is explicitly stated, is strongly supported, and is of great importance to improve the quality of care)
- Scientific soundness: measurement properties (the indicator is reliable, valid, and allows for stratification or case-mix-adjustment, if appropriate)
- Feasibility (the indicator is feasible in that the data source needed to implement the measure is available and accessible within the time frame for measurement, and the measure should have explicit and detailed specifications for the numerator and denominator)
- Importance of the measure (the indicator is important and relevant to knowledge users, has a high incidence or burden of illness, can be stratified for analyzing disparities in health, has potential for improvement, and is under the control of those providers whose performance is being measured)

Indicator inclusion criteria for rating in round 1 and round 2 were ≥75% of panelists' responses within the top tertile (scores of 7-9 on a 9-point Likert scale) for a given indicator, and a median composite score of 7 or greater was required. The indicator exclusion criteria were ≥75% of panelists' responses in the bottom tertile (scores of 1-3 on a 9-point Likert scale). Indicators meeting the inclusion criteria advanced to round 3 (ranking). Indicators meeting the exclusion criteria were automatically excluded. Where there was no consensus, indicators were included for rerating in round 2. Reminder emails were sent to panelists 2 weeks after the initial survey was sent in rounds 1, 2, and 3.

#### Delphi Round 2

Between the first and second rating rounds, a 3-hour virtual Delphi panel meeting was conducted to discuss the results of round 1, moderated by steering committee members. Before the meeting, each panel member received their rating scores for the indicators, along with median and IQRs of ratings of indicators based on round 1 results. During the meeting, novel indicators were also solicited based on perceived gaps from round 1. During the Delphi panel meeting, breakout groups were conducted for focused discussion on the novel indicators, indicators where there was no consensus, and to allow for more direct feedback from panelists regarding the indicators’ fidelity to the quality of virtual care. Round 2 rerating then progressed through the same approach as round 1.

#### Delphi Round 3

In round 3, panel members ranked the remaining indicators based on their importance in measuring the quality of virtual care. Due to the number of indicators with no consensus by round 3 of ranking, the modified Delphi approach was refined further to decrease the burden of Delphi panel members and minimize loss to follow-up of panelists. In round 3, panel members were to rank indicators from 1- “n” with 1 being most important to measuring quality within each domain of quality and “n” being least important within the specific Quintuple Aim domain. “N” represents the number of indicators within that specific quality domain. For example, if there were 11 indicators within patient experience, panel members ranked the remaining indicators 1 to 11, based on their perceived importance to measuring the quality of virtual care.

Results of the round 3 ranking exercise included medians and IQR of rankings. A Friedman test for the statistical significance was conducted to assess if differences in ranking were statistically significant. Where the “n” of indicators was robust enough, statistical significance of ranking differences was noted. Indicators were then reviewed in order of highest to lowest median ranking by an expert Steering Committee to enable the final selection of ranked indicators for inclusion.

### Ethical Considerations

This study was reviewed and approved through the Assessment Process for Quality Improvement Projects (APQIP) process at Women’s College Hospital (APQIP # 2021-0131-P). Funding for this project was provided by the Innovation Fund of the Alternative Funding Plan for the Academic Health Sciences Centres of Ontario (WCH-22-004).

## Results

### Curating Candidate Indicators

A scoping review was completed to assess existing quality indicators in the literature. Results are described in detail elsewhere [21]. Following the initial extraction, 245 indicators were extracted from 66 manuscripts. After removing duplicates, incomplete, or incoherent indicators (n=105), 140 quality indicators were then included for presentation to the Delphi panel.

### Pre-Delphi Survey

Beginning in June 2023, the pre-Delphi survey was sent to 189 members of CNDHE. A total of 33 members completed the survey (17.5% response rate). Results of the survey recommended adding cultural safety indicators when considering patient experience (n=3), digital literacy for providers (n=3), and equitable access to virtual care based on geographic status (n=2). The recommendations informed the generation of novel indicators between round 1 and round 2.

### Delphi Panel Process

#### Delphi Panelists

**Figure 1:**
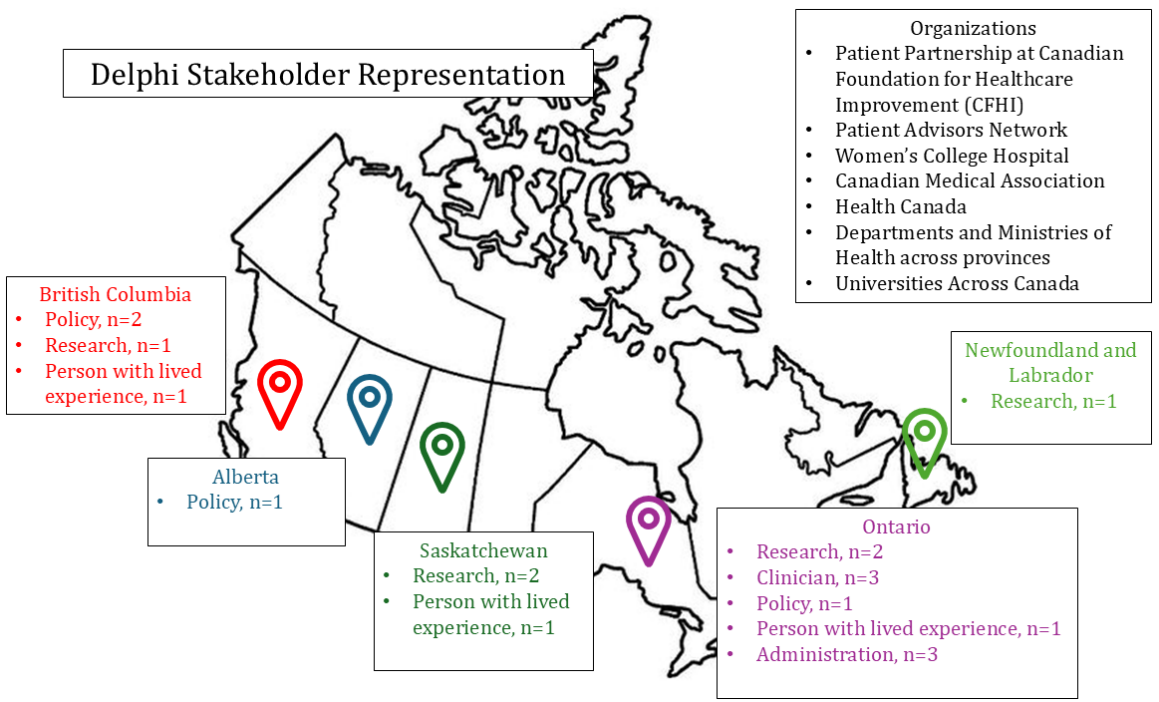
A map of modified Delphi panel member locations, as well as their primary affiliations. The panel was a diverse group of clinicians, researchers, and persons with lived experience from across Canada and urban or rural communities. N:19 panel members;.

A total of 35 people were invited to the Delphi introductory meeting, and 19 panelists contributed to all 3 rounds (54% response rate). Panelists were from Ontario (n=10); British Columbia (n=4); Saskatchewan (n=3); Alberta (n=1); and Newfoundland (n=1), and represented different organizations and backgrounds. Details on panelists are provided in Figure 1.

#### Delphi Round 1

**Figure 2:**
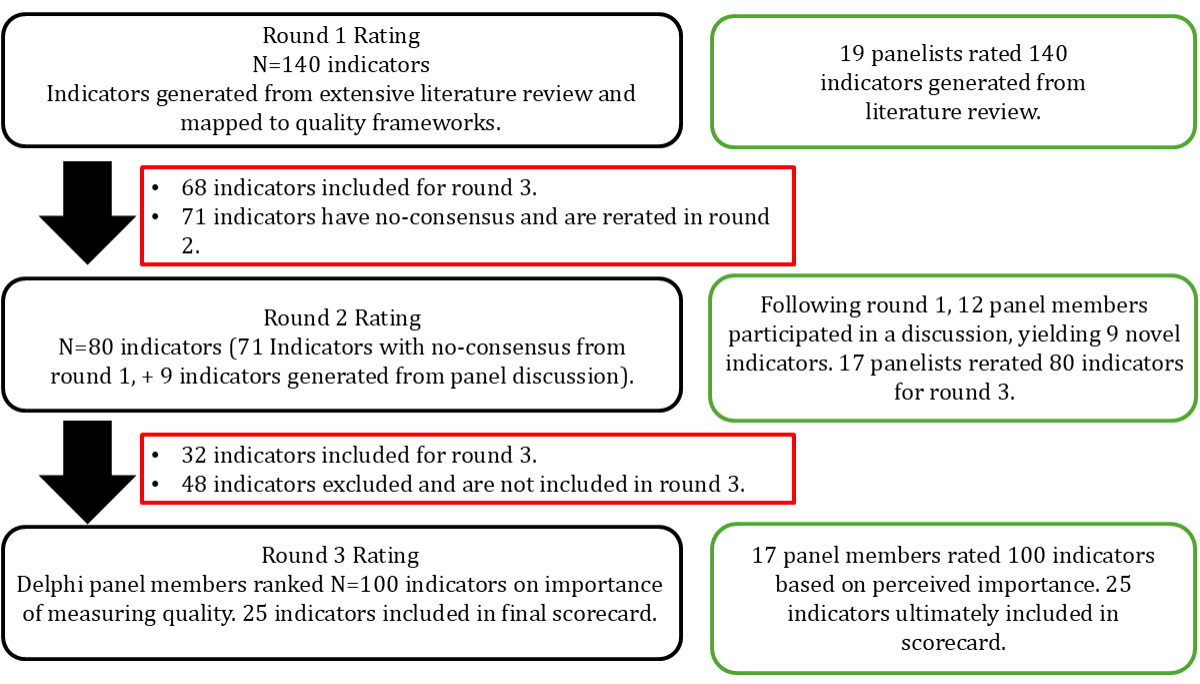
Summary of the Delphi process to select indicators of virtual care, across 3 rounds of rating, eventually leading to the final list of balanced scorecard for virtual care, which included 25 quality indicators.

Figure 2 provides an overview of the results of the 3-round modified Delphi panel process. In round 1, a total of 19 surveys were completed. There were 68 indicators included for the round 3 ranking, while none were excluded; 71 indicators had no consensus and required rerating in round 2. Table S3 in Multimedia Appendix 3 shows the results after round 1 of rating.

#### Delphi Round 2

Between round 1 and 2 rating exercises, a virtual Delphi panel meeting was held to discuss indicators that had not yet reached consensus (to either include or exclude) and to suggest novel indicators based on perceived gaps in current indicators. The meeting was attended by Delphi panel members who had completed round 1 (12/19, 63%). The meeting featured discussions on health equity and sustainability indicators, subcoded under patient experience and provider experience. Further discussion was also held regarding health system costs and patient-centered care, and breakout groups were used to discuss current and proposed indicators. The largest gaps identified included a limited focus on sustainability as well as health equity, which were addressed through the creation of novel indicators to be included in round 3. During this panel discussion, 9 novel indicators were developed by the Delphi panel to address perceived gaps in the indicators collected from the literature. These indicators were also informed by persons with lived experiences consultation and pre-Delphi survey.

Following the meeting, 19 surveys were distributed for rating the indicators without consensus, as well as 9 new indicators developed from the Delphi panel meeting, with 17 responses (17/19, 89%). In total, 32 indicators were included in the round 3 ranking, while 48 were excluded after round 2.

#### Delphi Round 3

In round 3 ranking, 17 surveys were sent to panel members to rank 100 indicators (68 indicators included from round 1 and 32 from round 2), with a 100% (17/17) Delphi panel response rate. The 2 panelists who did not complete the round 2 ranking were not contacted for round 3 and were considered lost to follow-up. Table S4 in Multimedia Appendix 4 includes the results of round 3. Indicators included in the final scorecard were based on their median ranking and IQR. Where applicable, the Friedman test was used to assess statistically significant differences in ranking between indicators within specific quality domains.

To finalize the set of quality indicators, the steering committee reviewed the list to ensure there was minimal repetition between ranked indicators, each one could be interpretable as an individual measure, and that gaps in indicators were addressed by Delphi panel-derived indicators. Ultimately, 3 novel indicators from the Delphi panel were included. These included (1) cultural safety and competency, (2) language preference, and (3) acknowledgment of a patient’s identity. The final list was further enhanced through review by content experts, including clinicians and leadership, who were external to the steering committee and not Delphi panelists. Aligned with the Quintuple Aim, there are 13 indicators on patient experience, 6 on provider experience, 2 on population health, 2 on health equity, and 2 on health system costs. The final list of indicators is presented in Table S1 in Multimedia Appendix 1.

## Discussion

### Principal Findings

Through a structured modified Delphi panel process, using broad knowledge user engagement that included persons with lived experiences in the co-design, a set of 25 quality indicators was curated to support organizations to evaluate the quality of ambulatory virtual care. The final set of indicators aligns with the Quintuple Aim framework, as well as the NAM quality domains of care, with the addition of sustainability to ensure a broad view of quality. Use of the set of indicators as part of a balanced scorecard can provide institutions with guidance on what measures to broadly assess the performance of virtual care within programs, to improve the quality of care provided. The scorecard is also designed to consider quality indicators, which are routinely underreported, such as equity and population health. For these reasons, the Delphi steering committee included indicators in the final scorecard that had minimal or no overlap with other included indicators, as well as indicators reflective of equity, health system costs, and population health to address gaps in the literature of virtual care evaluations [21]. The final scorecard reflects these decisions, as well as provides a more integrated framework for quality assessment compared to other measures currently used in practice.

### Implications for Practice

There have been calls for a more uniform approach to understanding the quality of care, particularly in the context of virtual encounters [32,33]. Quality indicators of care vary, sometimes significantly, between hospitals and institutions [17]. To the authors’ knowledge, an integrated framework for assessing the quality of virtual care has yet to be developed and validated. Existing tools, such as the telehealth usability questionnaire [34], are narrow in their focus and do not address the full scope of a virtual care program’s impact. By providing organizations with a balanced list of indicators across all relevant domains of quality within established frameworks, indicators will be relevant to knowledge users, helping to standardize approaches to measuring the quality of virtual care. This, in turn, can identify gaps in care delivery and inform rapid plan-do-study-act cycles of quality improvement, foundational to a learning health system (LHS) [34,35]. The aim is for the set of virtual quality indicators generated through this modified Delphi process to form the foundation for organizations across a health system to evaluate virtual care programs and as part of hybrid models of care.

Based on our previously completed literature review and extraction from quality indicators [21], there were several gaps identified by the Delphi panel in existing virtual care indicators. In response to this, the Delphi panel generated novel indicators, particularly centered on assessing equity and measuring the costs of virtual care. Lowering the barrier of entry for equitable virtual care, as well as ensuring virtual care programs are cost-effective, is a key next step in facilitating the transition from pilot models to standards of care.

This study has several strengths. The modified Delphi panel approach leveraged expertise from across Canada and from a diverse range of backgrounds, locations, and perspectives. There was minimal attrition of panel members through the 3 rounds of the modified Delphi process, with only 2 panel members being lost between rounds 1 and 2. There was strong involvement of persons with lived experiences in all phases of the project, from the steering committee to broad engagement in our Delphi panel process. Involving a person with lived experience in our steering committee helped shape and inform the study from its inception to analyzing and interpreting results, to being part of the decision-making process regarding the finalized list of quality indicators. Involving persons with lived experiences in research has multiple positive effects, including making end products relevant and pragmatic to implement [36]. We provided an opportunity for broad input from knowledge users to ensure our candidate list of indicators was comprehensive beyond our scoping review, which incorporated indicators from both published and grey literature to identify gaps and generate “novel” indicators not reflected in the existing literature. Finally, the methodological process was robust and relevant, as each indicator was rated using the validated AHRQ scale, and there was alignment of indicators to existing quality frameworks (Quintuple Aim and NAM domains of quality), which contributes to the relevance of using the scorecard to measure all relevant domains of quality of virtual care moving forward.

Systematic measurements of quality-of-care support more reliable comparisons of quality indicators across domains, jurisdictions, and contexts. This set of quality indicators aims to support institutions to align their goals and priorities and help diverse knowledge users communicate effectively regarding the quality of virtual care. Our next step is to test the feasibility and acceptability of this set of virtual care in practice and to support organizations to incorporate these indicators as part of existing quality scorecards. Indeed, while having a set of relevant quality indicators is a first step to evaluate the quality of virtual care, there is still uncertainty about how to use the scorecard in practice. It is likely that, for organizations to use the scorecard to its full scope, they will have to address issues such as evaluation resources (both infrastructure and human health resources), interoperability, and buy-in from organizational leadership. Understanding these barriers and facilitators is the next step in implementing the scorecard and pilot testing it within real-life health care contexts. Adaptations of the scorecard to local contexts will likely be anticipated based on local capacity considerations, and not all 25 indicators will be applicable in different settings. Further efforts will be directed to assess the feasibility of using this scorecard to drive quality improvement efforts and identify benchmarks.

### Limitations

There are limitations to the study to note. First, the heterogeneity of published quality indicators required transformation by the study team as they were extracted to ensure each indicator addressed a discrete quality consideration and could be measured. This adaptation process was done diligently, ensuring that fidelity to the quality indicator, as it was reported in the literature was maintained. In addition, published indicators could overlap and routinely fit within more than one quality domain; this was mitigated by dual-coding the indicators and resolving any discrepancy with a third reviewer. The Delphi panel methodology is opinion-based, there may be variations in recommendations based on the same evidence. In order to promote generalizability, we included a broad knowledge user group and ensured a rigorous rating and ranking process to combine scientific evidence with expert opinion to select the indicators using the modified Delphi panel methodology.

Quality of care is complex, and institutions can have challenges in effectively measuring certain quality domains, particularly equity and system costs [37]. Although we have presented a comprehensive list, some measures will be easier to feasibly measure than others, and further revisions may be required once the set is tested in practice. While the set of indicators provide guidance on what to measure, teams should ensure that the indicators reflect the needs of their individual setting and context. Each organization is unique, with its own intricacies and complexities, and consideration needs to be made to see what may be feasible in each setting and capacity level [38]. Some quality indicators are more technical than others; for example, indicators measuring system costs can be complex. To use these indicators, administrative personnel may be required, which may not be feasible at all organizations, particularly smaller, community clinics. Wherever possible, the steering committee tried to make the quality indicators accessible while maintaining fidelity to the extracted indicator and the Delphi rating and ranking process, and further streamlining is anticipated based on the learnings from our implementation process. In addition, 100 indicators were included for ranking for round 3, but the steering committee needed to be judicious to ensure a set of indicators that could be comprehensive to measure virtual care while balancing the number of indicators to include. Through the process, there were a number of quality indicators that may not have been included, to make the set of indicators more practical.

### Future Directions

Our next steps will focus on testing, implementing, and sharing the set of indicators. While our quality indicators have been rigorously curated, feedback from this implementation and dissemination phase will be essential to inform the feasibility and viability of the quality indicators in practice. One future direction is to understand how to embed the set of metrics within existing hospital scorecards as part of hybrid models of care. The next steps may include establishing benchmarks and continuously refining the scorecard through a learning health system approach [39,40]. The set of indicators will be a living document, constantly changing and updating to reflect best practices and the dynamic nature of digital health interventions.

### Conclusions

A balanced set of 25 quality indicators were rigorously developed through a modified Delphi panel process to assess virtual care with extensive knowledge of user engagement that incorporated person with lived experience. With inconsistencies in measuring the quality of virtual care across institutions, this set of indicators offers an evidence-informed approach to addressing this gap. This set of 25 quality indicators can be used as part of a structured approach aligned with the Quintuple Aim and the NAM domains of quality of care to ensure a comprehensive evaluation of the quality of virtual care. As virtual care becomes increasingly integrated into routine health care delivery, ensuring the quality and appropriateness of virtual encounters should be a key goal of health organizations and systems.

## Appendix

Multimedia Appendix 1 Balanced Scorecard for Virtual Care. 25 quality indicators were included from the Delphi process.

Multimedia Appendix 2 ACCORD (ACrutate COnsensus Reporting Document checklist) checklist.

Multimedia Appendix 3 Results of the rating after round 1 of Modified Delphi process.

Multimedia Appendix 4 Round 3 ranking (N=100 indicators).

## Abbreviations

ACCORD: ACrutate COnsensus Reporting Document checklist
AHRQ: Agency for Healthcare Research and Quality
APQIP: Assessment Process for Quality Improvement Projects
BSC: balanced scorecard
CLAC: Community Liaison Advisory Committee
CNDHE: Canadian Network for Digital Health Evaluation
JBI: Joanna Briggs Institute
LHS: learning health system
NAM: National Academies of Medicine
REDCap: Research Electronic Data Capture
