# Supplementary material for: Developing Balanced Quality Indicators for Monitoring Virtual Care in Ambulatory Care Environments: Modified Delphi Panel Process
Source: J Med Internet Res. 2025 Jun 16;27:e38657. doi: 10.2196/38657 (PMC12209721; doi:10.2196/38657)
Supplement: Multimedia Appendix 1 [file jmir_v27i1e38657_app1.pdf]

Supplementary Material S1 – Balanced Scorecard for Quality Virtual Care

*Denotes Indicator developed by Delphi Panel

Quintuple Aim	NAM Domain	Indicator Stem	Description	Actual Indicator	Adapted from References
**Patient Experience**	Effective	Consultation effectiveness	Measured by patient's understanding of the consultation, their confidence in the management of their condition, and their perception of the quality of the consultation.	**Measurement:**<br>Proportion of patients agreeing/disagreeing with survey statements<br>5-point Likert scale:<br>1=strongly disagree to<br>5=strongly agree<br><br>**Scoring:**<br>1) % of patients that reported they understood the reason for the consultation and felt better able to manage and understand their condition<br>2) % of patients that agreed with the statement "I was given adequate opportunity to express my opinion and ask questions during the consultation"<br>3) % of patients that reported the quality of the consultation as "excellent"	Joughin, A., Ibitoye, S., Crees, A., Shipway, D., & Braude, P. (2021). Developing a virtual geriatric perioperative Medicine Clinic: A Mixed Methods Healthcare Improvement Study. *Age and Ageing*, *50*(4), 1391–1396. https://doi.org/10.1093/ageing/afab066

Quintuple Aim	NAM Domain	Indicator Stem	Description	Actual Indicator	Adapted from References
**Patient Experience**	Effective	Communication quality	Measured by how easily and comfortably patients interact with their clinician using virtual care.	**Measurement:**<br>Survey items: I could easily talk to the clinician using virtual care; I could hear the clinician clearly using the virtual care; I feel comfortable communicating with the clinician using the virtual care; I felt I was able to express myself effectively.<br>5-point Likert scale: 1=strongly disagree to 5=strongly agree<br><br>**Scoring:**<br>Median and IQR for each item	Li MM, Rising KL, Goldberg EM. Transitioning to telehealth? A guide to evaluating outcomes. Health Policy Technol. 2022 Sep;11(3):100623. doi: 10.1016/j.hlpt.2022.100623. Epub 2022 Mar 27. PMID: 35369128; PMCID: PMC8957891.
**Patient Experience**		Technical quality	Evaluated through the patient's ability to clearly hear and comprehend the clinician's words, and to visually observe the clinician during the virtual consultation	**Measurement:**<br>Proportion of patients agreeing/disagreeing with survey statements<br>5-point Likert scale: 1=strongly disagree to 5=strongly agree<br><br>**Scoring:**<br>1) % of patients reported that they were able to hear and understand the clinician throughout the whole consultation<br>2) % of patients reported that they were able to see the clinician all of the time	Joughin, A., Ibitoye, S., Crees, A., Shipway, D., & Braude, P. (2021). Developing a virtual geriatric perioperative Medicine Clinic: A Mixed Methods Healthcare Improvement Study. *Age and Ageing*, *50*(4), 1391–1396. https://doi.org/10.1093/ageing/afab066

Quintuple Aim	NAM Domain	Indicator Stem	Description	Actual Indicator	Adapted from References
**Patient Experience**	Safety	Patient privacy	Measured by patient's perception of their privacy and body being safe when receiving virtual care	**Measurement:**<br>Proportion of patients agreeing/disagreeing with survey statements<br>Survey items: My privacy is protected during virtual care; I felt my body was safe during the virtual exam; I felt my privacy was safe during the virtual visit<br>5-point Likert scale: 1=strongly disagree to 5=strongly agree<br><br>**Scoring:**<br>1. % of patients reported feeling their privacy is protected during virtual care<br>2. % of patients agree with the statement "I felt my body was safe during the virtual exam"<br>% of patients agree with the statement "I felt my privacy was safe during the virtual visit"	Fraint, A., Stebbins, G. T., Pal, G., & Comella, C. L. (2019). Reliability, feasibility and satisfaction of telemedicine evaluations for Cervical Dystonia. *Journal of Telemedicine and Telecare*, *26*(9), 560–567. https://doi.org/10.1177/1357633x19853140<br><br>Adapted from this source.
**Patient Experience**	Sustainability	Preferences for future use	Assessed by the patient's willingness to opt for virtual visits in the future, including if it would save time or money	**Measurement:**<br>Survey items: In the future, I would like to use virtual care again; If I could save (travelling) time, I would prefer a virtual visit than a face-to-face visit; If I could save (travelling) money, I would prefer a virtual visit rather than a face-to-face visit<br>5-point Likert scale: 1=strongly disagree to 5=strongly agree<br><br>**Scoring:**<br>Total score across all items	Barsom, E.Z., Jansen, M., Tanis, P.J. *et al.* Video consultation during follow up care: effect on quality of care and patient- and provider attitude in patients with colorectal cancer. *Surg Endosc* **35**, 1278–1287 (2021). https://doi.org/10.1007/s00464-020-07499-3

Quintuple Aim	NAM Domain	Indicator Stem	Description	Actual Indicator	Adapted from References
**Patient Experience**	Sustainability	Recommendation (Net Promoter Score)	Measured by the patient's likelihood of recommending virtual care to their family and friends.	**Measurement:**<br>How likely are you to recommend this service to family and friends if they need similar care or treatment?<br>10-point Likert scale: 1 not at all likely 7 - 8 = passive to 9 -10 = extremely likely<br><br>**Scoring:**<br>Promoters (score 9-10)<br>Passives (score 7- 8)<br>Detractors (score 0-6)<br>To calculate the NPS, percentage of Detractors is subtracted from the percentage of Promoters.<br>NPS > 0: Promoters are higher than Detractors<br>NPS = 0: Equal number of Promoters and Detractors<br>NPS<0: Detractors are higher than Promoters	Alismail A, Schaeffer B, Oh A, Hamiduzzaman S, Daher N, Song HY, Furukawa B, Tan LD. The Use of the Net Promoter Score (NPS) in an Outpatient Allergy and Pulmonary Clinic: An Innovative Look into Using Tablet-Based Tool vs Traditional Survey Method. Patient Relat Outcome Meas. 2020 May 19;11:137-142. doi: 10.2147/PROM.S248431. PMID: 32547281; PMCID: PMC7245467.

Quintuple Aim	NAM Domain	Indicator Stem	Description	Actual Indicator	Adapted from References
**Patient Experience**	Composite	Telemedicine Usability Questionnaire (TUQ)	The Telemedicine Usability Questionnaire (TUQ) evaluates the usability of telehealth implementation and services and is designed to be a comprehensive questionnaire that covers all usability factors, including usefulness, ease of use, effectiveness, reliability, and satisfaction.	**Measurement:** 7-point Likert scale: strongly disagree to strongly agree (18 items total)<br><br>**Scoring:** Overall mean of all items, higher score indicates higher satisfaction	Blanco Terés, L., Cerdán Santacruz, C., García Septiem, J., Maqueda González, R., Lopesino González, J. M., Correa Bonito, A., & Martín-Pérez, E. (2021). Patients' perceived satisfaction through telephone-assisted Tele-consultation during the SARS-COV-2 pandemic period: Observational single-centre study at a tertiary-referral colorectal surgery department. *Surgical Innovation*, *29*(1), 35–43. https://doi.org/10.1177/15533506211008053
**Patient Experience**	Timely	Timely access to care	Refers to the ability of individuals to obtain necessary healthcare services promptly when needed.	**Measurement:** Average wait time per case	Khairat S, Lin X, Liu S, Man Z, Zaman T, Edson B, Gianforcaro R. Evaluation of Patient Experience During Virtual and In-Person Urgent Care Visits: Time and Cost Analysis. J Patient Exp. 2021 Jan 12;8:2374373520981487. doi: 10.1177/2374373520981487. PMID: 34189260; PMCID: PMC8205332.<br><br>Adapted from this source.

Quintuple Aim	NAM Domain	Indicator Stem	Description	Actual Indicator	Adapted from References
**Patient Experience**	Equitable	Cost saved by patient	Ability of virtual care to reduce healthcare-related expenses	**Measurement:**<br>Did virtual care save you any money? For travel; accommodation; taking time off work or school; requiring caregiver/spouse/partner present during the visit taking time off work or school.<br><br>**Scoring:** Scored across five categories:<br>1 No money saved<br>2 Saved $10-$60<br>3 Saved $61-$120<br>4 Saved $121-$250<br>5 Saved $250+	Bate, N. J., Xu, S. C., Pacilli, M., Roberts, L. J., Kimber, C., & Nataraja, R. M. (2021). Effect of the COVID-19 induced phase of massive telehealth uptake on end-user satisfaction. *Internal Medicine Journal*, *51*(2), 206–214. https://doi.org/10.1111/imj.15222<br><br>Adapted from this source.
**Patient Experience**		Access to care	Ability of virtual care to improve access to care and save time travelling to a hospital or clinic	**Measurement:**<br>2 items: virtual care saves me time travelling to a hospital or clinic; virtual care improves my access to healthcare services. 7-point Likert scale: 1= strongly disagree to 7=strongly agree.<br><br>**Scoring:**<br>Median and inter-quartile range (IQR) for each item	Cottrell M, Burns CL, Jones A, Rahmann A, Young A, Sam S, Cruickshank M, Pateman K. Sustaining allied health telehealth services beyond the rapid response to COVID-19: Learning from patient and staff experiences at a large quaternary hospital. J Telemed Telecare. 2021 Dec;27(10):615-624. doi: 10.1177/1357633X211041517. PMID: 34726993; PMCID: PMC8564219.

Quintuple Aim	NAM Domain	Indicator Stem	Description	Actual Indicator	Adapted from References
**Patient Experience**	Equitable	Accessibility	Accessibility refers to the design and provision of (virtual care) services, environments, and products in a way that ensures they can be used by everyone, including people living with disabilities.	**Measurement:**<br>Proportion of people responding positively to the statements<br>"I am able to receive care in my preferred language during my virtual visit"<br>"I am able to access assisted supports/technologies as needed during my virtual visit"<br><br>**Proposed scoring:**<br>5-point Likert scale - 1= strongly disagree to 5=strongly agree	Generated by Delphi panel members<br><br>Canadian Institute for Health Information. *Measuring Cultural Safety in Health Systems: Addendum – List of Indicators.* Ottawa, ON: CIHI; 2021.<br><br>Adapted from this source for the context of virtual care.
**Patient Experience**		Acknowledgement of identity*	Perceptions of (virtual) care quality based on whether patients feel that their racial and/or ethnic identity, sexual identity, culture and community are acknowledged	**Measurement:**<br>Proportion of people who: Felt that their identity was positively viewed during their virtual care visit by (1) health providers/professionals; (2) staff; Reported that they had the resources, supports and spaces available to support their holistic wellness; Felt that the diversity of cultures was understood by the organization; Reported that their health care providers were open to hearing about traditional/alternative medicine; Reported that health care providers were knowledgeable about available health services in their community<br>**Proposed scoring:**<br>5-point Likert scale: 1= strongly disagree to 5=strongly agree	Generated by Delphi panel members<br><br>Canadian Institute for Health Information. *Measuring Cultural Safety in Health Systems: Addendum – List of Indicators.* Ottawa, ON: CIHI; 2021.<br><br>Adapted from this source for the context of virtual care.

Quintuple Aim	NAM Domain	Indicator Stem	Description	Actual Indicator	Adapted from References
**Patient Experience**	Patient Centeredness	Patient engagement and self-management	Measured by patient's access to health information and the sense of control they feel over their health and healthcare decisions.	**Measurement:**<br>Survey items: I have all the information I need to manage my health and health care; I am confident in working with my care team to manage my health and health care; I feel in control of my health and health care (such as taking part in decisions or following through on any medication, treatment, or health routine); I am able to achieve my long-term health and health care goals (such as being self-reliant, living longer and better, or knowing that my family and friends can depend on me)<br>5-point Likert scale: 1=strongly disagree to 5=strongly agree<br>**Scoring:**<br>Total score across all items	Garvin LA, Simon SR Prioritizing Measures of Digital Patient Engagement: A Delphi Expert Panel Study J Med Internet Res 2017;19(5):e182
**Provider Experience**	Efficient	Clinician and clinic efficiency	Increased clinician and clinic efficiency associated with wait times, no shows and cancellations of appointments	**Measurement:**<br>Median wait time per patient; number of no-shows/cancellations<br><br>**Scoring:**<br>N/A	Watts KA, Malone E, Dionne-OdomJN, et al. Can you hear me now? Improving palliative care access through telehealth. Res Nurs Health. 2021;44:226–237.https://doi.org/10.1002/nur.22105

Quintuple Aim	NAM Domain	Indicator Stem	Description	Actual Indicator	Adapted from References
**Provider Experience**	Efficient	Interoperability & portability*	Degree of interoperability and ability to integrate information from external organizations to support virtual care delivery	**Measurement:** Percentage of clinicians concerned with current integration of heterogeneous platforms with EMR during a virtual care encounter<br><br>**Scoring:** 5-point Likert scale: 1=strongly disagree to 5=strongly agree	Generated by Delphi panel members<br><br>Mohammed HT, Hyseni L, Bui V, Gerritsen B, Fuller K, Sung J, Alarakhia M. Exploring the use and challenges of implementing virtual visits during COVID-19 in primary care and lessons for sustained use. PLoS One. 2021 Jun 24;16(6):e0253665. doi: 10.1371/journal.pone.0253665. PMID: 34166441; PMCID: PMC8224904.<br><br>Adapted from this source.
**Provider Experience**	Safety	Clinician burnout*	Clinicians' perceptions of how "burnt out" they feel related to their use of virtual care. Burnout is an occupational condition characterized by emotional exhaustion, depersonalization, and a low sense of personal accomplishment.	**Measurement:** Survey question: Overall, based on your definition of burnout, how would you rate your level of burnout related to using virtual care and/or digital health tools? Responses are scored on a five-category ordinal scale, where 1 = "I enjoy my work. I have no symptoms of burnout;" 2 = "Occasionally I am under stress, and I don't always have as much energy as I once did, but I don't feel burned out;" 3 = "I am definitely burning out and have one or more symptoms of burnout, such as physical and emotional exhaustion;" 4 = "The symptoms of burnout that I'm experiencing won't go away. I think about frustration at work a lot;" and 5 = "I feel completely burned out and often wonder if I can go on. I am at the point where I may need some	Generated by Delphi panel members<br><br>Dolan, E. D., Mohr, D., Lempa, M., Joos, S., Fihn, S. D., Nelson, K. M., & Helfrich, C. D. (2014). Using a single item to measure burnout in primary care staff: A psychometric evaluation. *Journal of General Internal Medicine*, *30*(5), 582–587. https://doi.org/10.1007/s11606-014-3112-6

				changes or may need to seek some sort of help."<br><br>**Scoring:**<br>Dichotomized as ≤2 (no symptoms of burnout) vs. ≥3 (1 or more symptoms)	

Quintuple Aim	NAM Domain	Indicator Stem	Description	Actual Indicator	Adapted from References
**Provider Experience**	Sustainable	Technology infrastructure	Sustainable delivery of virtual care needs systems with technology and knowledge to consistently and reliably review patient data and alerts and to develop appropriate algorithms to respond to patient data in a manner that improves patient outcomes.	**Measurement:**<br>Presence of: system reliability, data quality,<br>storage, transmission, system speed, interoperability, user interface, information security<br><br>**Scoring:**<br>N/A	Chang, H. (2015). Evaluation framework for telemedicine using the Logical Framework Approach and a fishbone diagram. *Healthcare Informatics Research*, *21*(4), 230. https://doi.org/10.4258/hir.2015.21.4.230<br><br>Adapted from this source.
**Provider Experience**		Future preference for virtual care	Willingness and preference to conduct future appointments via virtual visits whenever feasible and clinically relevant.	**Measurement:**<br>To what extent do you agree with the following statement about virtual care: If given the option, I would favor having a future virtual care appointment with this patient; If given the option, I would prefer future virtual care appointment whenever possible.<br>Measurement: 10-<br>point Likert scale - 1=highly disagree, 10= highly agree<br><br>**Scoring:**<br>Median scores	Hanson, R. E., Truesdell, M., Stebbins, G. T., Weathers, A. L., & Goetz, C. G. (2019). Telemedicine vs office visits in a Movement Disorders Clinic: Comparative satisfaction of physicians and patients. *Movement Disorders Clinical Practice*, *6*(1), 65–69. https://doi.org/10.1002/mdc3.12703<br><br>Adapted from this source.

Quintuple Aim	NAM Domain	Indicator Stem	Description	Actual Indicator	Adapted from References
**Provider Experience**	Effective	Ability to evaluate and treat patient	Assessment of the provider's capability to virtually manage and care for the patient comprehensively.	**Measurement:** Opinion on the following statements: How well could you evaluate/examine the patient? How well could you treat the patient?; Overall how well could you assess/treat/check the patient? 5-point Likert scale: Very bad to Very good<br><br>**Scoring:** Proportion responding Good/Very good	Buvik et al. BMC Health Services Research (2016) 16:483 DOI 10.1186/s12913-016-1717-7
**Population Health**	*No NAM Domain*	Self-rated health status	Self-rated health status for patients during or after receiving virtual care	**Measurement:** EQ visual analogue scale (EQ-5D-VAS) - a vertically graded line from 0-100<br><br>**Scoring:** Higher score	Augestad, K M; Sneve, A M; Lindsetmo, R-O. The British journal of surgery / 2020;107(5):509-518 England 2020/

Quintuple Aim	NAM Domain	Indicator Stem	Description	Actual Indicator	Adapted from References
**Population Health**	*No NAM Domain*	Adverse events	The presence of serious and non-serious adverse events, defined as any clinical change or illness suspected to be related to virtual care.	**Measurement:**<br><br>Survey question:<br>Indicate if the patient has been involved in a virtual care encounter suspected to have led to any of the following adverse events related to care being provided virtually:<br><br>1. Misdiagnosis<br><br>Emergency situations where there was an inability to respond	Adverse events related to virtual care. https://cpsp.cps.ca/uploads/studies/Protocol_Adverse_events_related_to_virtual_care_Nov_7_2022_FINAL.pdf<br><br>Adapted from this source for the context of virtual care.
**Health Equity**	*No NAM Domain*	Equal access to care	Virtual care has a potential wide reach and can transcend diverse population characteristics, demonstrated by participants varied diagnoses, gender, and origin from different countries†	**Measurement:**<br>Percentage of patient accessing virtual care stratified by demographics age, gender, income (neighbourhood quintile via postal code), geography (distance from hospital via postal code)<br><br>**Scoring:** N/A<br><br>† age, gender, race, postal code especially in rural/low- and middle-income areas	Allen Watts, Kristen; Malone, Emily; Dionne-Odom, J Nicholas; Currie, Erin; Hicks, Jennifer; McCammon, Susan; Tucker, Rodney O.; Elk, Ronit; Bakitas, Marie; Wallace, Eric<br>Research in nursing & health / 2021;44(1):226-237<br>United States NLM (Medline) 2021 /DOI: 10.1002/nur.22105<br><br>Adapted from this source for the context of virtual care.

Quintuple Aim	NAM Domain	Indicator Stem	Description	Actual Indicator	Adapted from References
**Health Equity**	*No NAM Domain*	Organizational Infrastructure	Level of access to the internet has implications on the patient's experience of and ability to access virtual healthcare services	**Measurement:**<br>Level of access to the internet measured by: 1) access to the internet (yes/no); 2) broadband is sufficient for activities in the view of system users (continuous provisions/regular provision/never worked)<br><br>**Scoring:** Percentage of responses from each category	Dos Santos, A. de, Mata-Machado, A. T., Melo, M. do, Fonseca Sobrinho, D., Araújo, L. L., Silva, É. A., Lima, A. M., Abreu, D. M., & Rocha, H. A. (2019). Implementation of telehealth resources in primary care in Brazil and its association with quality of care. *Telemedicine and E-Health*, *25*(10), 996–1004. https://doi.org/10.1089/tmj.2018.0166
**Health System Costs**	*No NAM Domain*	Healthcare utilization after virtual visit	Contacts with healthcare system following a virtual visit/consultation	**Measurement:**<br>Hospitalization rates: 30-day hospitalizations and/or 30-day emergency department visits after virtual visit/contact<br>Length of hospital stay after prior virtual visit<br><br>**Scoring:**<br>% of patients hospitalized and/or visit the emergency department after virtual visits compared to patients without prior virtual visits.<br><br>Mean hospital length of stay after prior virtual visit	Rawal S, Kwan JL, Razak F, Detsky AS, Guo Y, Lapointe-Shaw L, Tang T, Weinerman A, Laupacis A, Subramanian SV, Verma AA. Association of the Trauma of Hospitalization With 30-Day Readmission or Emergency Department Visit. JAMA Intern Med. 2019 Jan 1;179(1):38-45. doi: 10.1001/jamainternmed.2018.5100. PMID: 30508018; PMCID: PMC6583419.<br><br>Adapted from this source for the context of virtual care.

Quintuple Aim	NAM Domain	Indicator Stem	Description	Actual Indicator	Adapted from References
**Health System Costs**	*No NAM Domain*	Organizational costs	Costs for virtual care delivery include broadband affordability and quality, technical support for device or connection issues, additional equipment, and the clinical space.	**Measurement:** Costs associated with telehealth structure including: broadband services with appropriate bandwidth, availability of a technical support team to help address any device or connection issues, availability of additional equipment, appropriate clinical space	Haleem A, Javaid M, Singh RP, Suman R. Telemedicine for healthcare: Capabilities, features, barriers, and applications. Sens Int. 2021;2:100117. doi: 10.1016/j.sintl.2021.100117. Epub 2021 Jul 24. PMID: 34806053; PMCID: PMC8590973.
