# Supplementary material for: Developing Balanced Quality Indicators for Monitoring Virtual Care in Ambulatory Care Environments: Modified Delphi Panel Process
Source: J Med Internet Res. 2025 Jun 16;27:e38657. doi: 10.2196/38657 (PMC12209721; doi:10.2196/38657)
Supplement: Multimedia Appendix 3 [file jmir_v27i1e38657_app3.pdf]

Supplementary Material Table 1: Results of the rating after Round 1 of Modified Delphi Process

**Indicator Category**	**Included**	**Excluded**	**No Consensus**
**Total**	**69**	**0**	**71**
**Patient Experience**	**39**	**0**	**38**
Composite	1	0	7
Effective	5	0	13
Efficient	7	0	1
Equitable	10	0	4
Patient-centered	11	0	7
Safety	4	0	1
Sustainable	0	0	4
Timely	1	0	4
**Provider Experience**	**17**	**0**	**15**
Composite	3	0	0
Effective	4	0	3
Efficient	5	0	2
Patient-centered	0	0	5
Safety	2	0	0
Sustainable	3	0	5
**Health Equity**	**4**	**0**	**2**
**Health System Cost**	**2**	**0**	**9**
**Population Health Outcomes**	**7**	**0**	**4**
