# Supplementary material for: Developing Balanced Quality Indicators for Monitoring Virtual Care in Ambulatory Care Environments: Modified Delphi Panel Process
Source: J Med Internet Res. 2025 Jun 16;27:e38657. doi: 10.2196/38657 (PMC12209721; doi:10.2196/38657)
Supplement: Multimedia Appendix 4 [file jmir_v27i1e38657_app4.pdf]

Supplementary Material Table S4 – Round 3 Ranking (N=100 Indicators)

Table 4: Round 3 results of Delphi panel. Remaining indicators were ranked on scale of 1-*n*, where *n* was the number of indicators under review. Where appropriate, Friedman's test of statistical significance was completed to evaluate if the difference in ranking was statistically significant.

Quintuple Aim	NAM Domain	Indicator Stem	Median	IQR	Friedman's Test p-value
Patient Experience	Composite	Telemedicine Usability Questionnaire (TUQ)	1	1	N/A (2 indicators)
		Client Satisfaction Questionnaire (CSQ)	2	1	
	Effective	Consultation effectiveness	3	3	0.000531
		Satisfaction with care	3	2	
		Overall quality of visit	4	4	
		Communication quality	5	2	
		Comparison to in-person care	6	4	
		Perception of visit	6	3	
		Preference for virtual appointment compared to face-to-face	6	4	
		Feasibility of virtual care	7	3	
		Satisfaction with application	7	4	
	Efficient	Barriers to virtual consultation	5	4	0.1913
		Perceived convenience of virtual visits	4	3	
		Perceived usefulness	3	2	
		Perception of ease of use	3	4	

		Technical quality	5	4	
		Usability	4	2	
		Visit time	5	4	
	Equitable	Access to care	1	1	0.00000002319
		Equitability and accessibility	3	3	
		Cost saved by Patient	5	6	
		Culture safety	5	4	
		Patient burden	6	3	
		Access to information	6	4	
		Acknowledgement of identity	6	5	
		Language preference	6	7	
		Patient savings	9	2	
		Costs saved by patient	9	3	
		Time saved by appointment	9	6	
		Patient Assessment of Communication during Telemedicine (PACT)-Convenience of Visit subscale	10	5	
	Patient-centered	Patient engagement	3	4	
		Patient satisfaction and value	4	4	
		Increased self-management	6	6	
		Standard of care	6	7	
		Communication preference	7	4	
		Satisfaction with care	7	7	
		Suitability of video visit	8	5	
		Practice guidelines	8	8	
		Acceptability of remote monitoring	9	7	
		Set-up convenience	9	6	

		Perception of virtual care	9	5	
		Telehealth satisfaction scale (TeSS)	9	7	
		Perception of virtual communication	10	6	0.006597
	Safety	Patient privacy	3	2	0.3401
		Perception of safety	2	2	
		Patient Assessment of Communication during Telemedicine (PACT)-Physician's Clinical Competence and Skills subscale	3	2	
		Access to medical information	2	2	
	Sustainable	Preferences for future use	2	2	0.056
		Program sustainability	2	1	
		Data Access and Portability	3	1	
	Timely	Provider and clinic efficiency	3	2	N/A (2 indicators)
		Timely access to care	2	1	
Provider Experience	Composite	Telemedicine Usability Questionnaire (TUQ)	2	2	0.6296
		Telemedicine Satisfaction Questionnaire (TSQ)	2	1	
		Physician-Patient Satisfaction Questionnaire (P-PSQ)	2	2	
	Effective	Quality of Service	3	3	
		Satisfaction with visit	4	3	

		Ability to evaluate and treat patient	2	2	
		Ability to treat patient	3	2	
		Overall ability to assess/check/treat patients	3	2	0.3084
	Efficient	Barriers to using telehealth	3	4	0.8338
		Time effectiveness	4	2	
		Visit effectiveness	3	2	
		Provider workflow	3	3	
		Provider workload	4	2	
		Process efficiency	4	4	
	Safety	Patient safety	1	1	N/A (2 indicators)
		Clinician burnout	2	1	
	Sustainable	Organizational changes	2	4	0.0003415
		Technology infrastructure	3	3	
		Future preference for virtual care	4	5	
		Interprofessional cooperation	4	3	
		Policies and regulations	5	3	
		Provider behaviour	5	3	
		Provider data access	6	5	
		Missed appointments	7	2	
Health Equity		Equal access to care	2	2	
		Equitability and accessibility	2	2	
		Inclusion of patient voice	4	1	
		Structure	5	4	
		Infrastructure	5	1	
		Online accessibility	5	2	

	Travel cost	6	2	0.00009997
Health System Cost	Structural costs	5	4	0.1599
	Healthcare utilization-emergent	4	2	
	Healthcare utilization-impatient	4	2	
	Healthcare utilization-general	3	2	
	Incremental cost-utility ratio (ICUR)	2	2	
	Incremental cost-effectiveness ratio (ICER)	3	4	
Population Health Outcomes	Adverse events	5	6	0.1966
	Healthcare utilization	5	7	
	Concordance with care guidelines	5	7	
	Accuracy	7	5	
	Engagement Post-treatment	5	3	
	Quality of Service	6	7	
	Process -of-care measures	6	2	
	Self-rated health status	8	3	
	Time effectiveness	8	5	
	Timely care	4	3	
	Interoperability and portability	7	4	
